# The effects of racism and resilience on Black stroke- survivor quality of life: Study protocol and rationale for a mixed-methods approach

**DOI:** 10.3389/fneur.2022.885374

**Published:** 2022-08-10

**Authors:** Mary F. Love, Andrea Nicole Brooks, Sonya D. Cox, Munachi Okpala, Gail Cooksey, Audrey Sarah Cohen, Anjail Z. Sharrief

**Affiliations:** ^1^College of Nursing, University of Houston, Houston, TX, United States; ^2^Department of Neurology, McGovern Medical School, The University of Texas Health Science Center Houston, Houston, TX, United States

**Keywords:** stroke, stress, resilience, racism, quality of life

## Abstract

**Introduction:**

Stroke, a life-threatening stressor, often negatively impacts stroke-survivor (SS) quality of life (QoL). Annual age-adjusted incidence and death rates for stroke are significantly higher among Black Americans than among White Americans. Racism, a significant stressor, occurs at structural, cultural, and interpersonal levels and contributes to health disparities for Black SS. Resilience, a dynamic process of positive adaptation to significant stress, is impacted by factors or resources both internal and external to the individual. This study aims to examine the effects of experiences of racism and resilience on Black SS QoL during early stroke recovery. This article presents the study protocol.

**Methods and analyses:**

This will be a prospective observational mixed-methods study. Black community-dwelling adults who are within 4 weeks of a stroke will be eligible for inclusion. Baseline measures will include the exposure variables of experiences of racism and resilience. Covariates measured at baseline include sociodemographic variables (age, sex, marital status, education, income, health insurance, employment status, number of people in household, residential address), clinical variables (date and type of stroke, inferred Modified Rankin Scale, anxiety and depression screening), and psychosocial variables (COVID-19 stress, perceived stress, mindfulness). The outcome variable (QoL) will be assessed 6-months post-stroke. Multiple-level linear regression models will be used to test the direct effects of experiences of racism, and the direct and indirect effects of resilience, on QoL. Qualitative data will be collected *via* focus groups and analyzed for themes of racism, resilience, and QoL.

**Discussion:**

Racism can compound the stress exerted by stroke on Black SS. This study will occur during the COVID-19 pandemic and in the aftermath of calls for social justice for Black Americans. Experiences of racism will be measured with instruments for both “everyday” discrimination and vigilance. Sociodemographic variables will be operationalized to assess specific social determinants of health that intersect with structural racism. Because of the long-standing history of racism in the United States of America (USA), cultural influences and access to resources are central to the consideration of individual-level resilience in Black SS. Study results may inform the development of interventions to support Black SS QoL through enhanced resilience.

## Introduction

The worldwide prevalence of stroke is estimated at 80.1 million people, and ~7.6 million Americans currently live in the aftermath of stroke (1, 2). Stroke is an acute, life-threatening event that exerts considerable stress on stroke survivors. Psychosocial stress (e.g., stress related to work, home, finances, major life events, and other stressors) has been identified as both a risk factor for stroke (3, 4) and a consequence of stroke (4). This bidirectional relationship between stroke and stress operates through various biological (dysregulation of the sympathetic nervous system and hypothalamic-pituitary-adrenal axis), behavioral (smoking, unhealthy diet, sedentary lifestyle) and psychological (anxiety, depression, post-traumatic stress) pathways to impact both physical and mental health, resulting in negative impacts on SS outcomes and recovery (4). Psychosocial factors, which reflect how individuals respond to stress, can also influence SS QoL (5). The World Health Organization defines quality of life as “an individual's perception of their position in life in the context of the culture and value systems in which they live and in relation to their goals, expectations, standards and concerns” (6). Stroke survivors experience lower QoL immediately post-stroke, with most of the recovery in QoL levels occurring in the first 6 months after stroke (7). In the first year post-stroke, social factors of female sex and Black race have been associated with lower levels of QoL, while being married has been associated with higher levels of QoL (8).

Post-stroke care in the USA has typically focused on functional, cognitive, and communicative impairments, and more recently on secondary stroke prevention, resulting in gaps in recognizing and treating psychosocial factors related to stroke (9, 10). These gaps can result in unmet psychosocial needs, which can impact quality of life and adherence to stroke risk-factor management strategies (11). Unmet psychosocial needs are particularly relevant for those with milder stroke, characterized by SS with limited impairments who may be discharged from the hospital ≤ 4 days post-stroke, and may receive minimal services post discharge (12, 13). Recent medical advances in stroke care (e.g., mobile stroke units, thrombolysis, mechanical thrombectomy), which have been associated with reductions in stroke severity, will likely increase the prevalence of milder stroke (10, 14).

Significant health disparities for stroke are evident in the (USA) for Black Americans compared to White Americans. Healthy People 2020 defines a health disparity as “a particular type of health difference that is closely linked with social, economic, and/or environmental disadvantage” (15). The overall incidence of stroke in the USA is higher for Black adults than White adults (IRR 1.51, 95% CI 1.26–1.81), with an even higher incidence for those between 45 and 54 years of age (IRR 4.02, 95% CI 1.23–13.11) (16). Females have a higher lifetime risk for stroke than males (2), and Black females in the USA have a higher risk for stroke than White females (HR, 1.47, 95% CI 1.33–1.63) that persists after adjustment for socioeconomic status and stroke risk factors for those aged 50 to 60 years (HR, 1.76, 95% CI 1.09–2.83) (17). Black stroke survivors (SS) in the USA tend to receive lower quality of post-stroke care than White SS (10). These disparities are directly related to racism, which operates at structural, cultural, and interpersonal levels in the USA (18, 19).

Racism is a significant stressor for Black SS that compounds the stress exerted by stroke. Structural racism refers to the societal structures and policies which have been normalized over time and which reduce access to desirable resources and opportunities for Black and other people of color in comparison to White people (18, 20). Structural racism, a social determinant of health (SDOH), is manifested through other SDOH including unequal access to health care, quality education, healthy foods, adequate housing, transportation, safe neighborhoods and employment opportunities (18, 21). Cultural racism refers to embedded beliefs and norms in the larger culture regarding the superiority of White people in comparison to Black people, leading to negative attitudes and beliefs that devalue these populations (20), while interpersonal racism refers to prejudice and discrimination that occurs at the individual level (18, 19).

Proposed pathways through which racism negatively impacts individual mental and physical health outcomes include increased stress responses (biological and psychological) and altered health behaviors (19, 22). Williams et al. (20) conducted a review of literature reviews and meta-analyses regarding relationships between racism and health, with a focus on self-reported racial discrimination, mental and physical health, health behaviors, and health care utilization. Racial discrimination was consistently associated with poorer mental health outcomes (e.g., depression, anxiety, psychological distress, lower wellbeing). For physical health outcomes, significant associations were seen between racial discrimination and obesity, hypertension, biomarkers for HPA axis reactivity and inflammation, and lower telomere length (comparing Black people to White people). Health behaviors of alcohol use (but not alcohol abuse), smoking and poor sleep were also associated with racial discrimination. Racial discrimination was also associated with higher levels of mistrust in health care workers and systems, and lower levels of satisfaction with care and patient-provider communication (20).

Resilience can be considered a dynamic process of positive adaptation to significant stress, adversity, or trauma, such as stroke (23). Rather than being a static trait, resilience is a dynamic process that is influenced by different contexts experienced throughout an individual's life (24) or throughout the acute and chronic phases of medical diagnoses such as stroke (25). Panter-Brick uses a cultural perspective to describe resilience as constructing meaning from experiences of adversity and stress, a relevant viewpoint in consideration of the stressors faced by Black SS (26). Resilience of individuals (such as stroke survivors) is affected by factors or resources at the individual, interpersonal, and structural (community/societal) levels (25). These factors, which can be further characterized as either protective or vulnerability/risk factors (26), interact to shape the individual's response to stressful experiences (25, 26).

Individual-level factors or resources include psychological, behavioral, and biological factors. IIndividual-level factors that support resilience in adults include positive emotion, purpose and meaning in life (27, 28), mindfulness (29), spirituality, and mood clarity (30). Social support, an interpersonal-level factor, is also a protective factor for adult resilience (31). Among those with cardiovascular disease, resilience has demonstrated significant associations with higher quality of life in patients with coronary artery disease (32), acute myocardial infarction (33), and stroke (34). Resilience in stroke survivors has shown inverse correlations with individual-level factors of depression (34, 35) and anxiety (34), and positive associations with physical independence (34), self-efficacy (36), and religion (36). Among SS, higher education and having medical insurance (35) have been associated with higher resilience, while being the main source of income and having lower income (34, 36) have been associated with lower resilience.

Studies regarding the effects of racism and/or resilience on quality of life for Black SS are largely absent. The aim of this article is to present the study protocol. The purpose of this study will be to examine the direct effect of experiences of racism, and the direct and indirect effects of resilience, on Black SS QoL in the first 6 months of stroke recovery. We will also explore the qualitative experiences of racism and resilience in our sample.

## Methods and analysis

### Study design and rationale

This is a prospective observational study in which a subset of the sample will also participate in a focus group interview, resulting in an embedded mixed-methods study design. Mixed-methods studies combine the strengths of qualitative and quantitative methods, resulting in a more robust design to offset weaknesses of each method (37). We will use a qualitative descriptive approach for the qualitative portion of the study. This approach focuses on interpreting findings while staying close to literal or straight description that fits the data (38).

This study will be conducted in a large urban area is the southeastern USA. The target population consists of community-dwelling stroke survivors in the first 6 months of stroke recovery who are discharged either directly home or to inpatient stroke rehabilitation for no more than 4 weeks. Participants will be recruited during the first post-stroke visit at the university-based stroke clinic affiliated with the admitting hospital, a Comprehensive Stroke Center. The stroke clinic visit will occur either in-person or virtually. Community-dwelling adults (age ≥ 18 years) who self-identify as a Black person and have a recent (within 4 weeks) diagnosis of ischemic stroke, hemorrhagic stroke or transient ischemic attack (TIA) will be eligible for study participation. Additional inclusion criteria include the ability to speak and read English. Stroke survivors will be excluded from the study if they have severe aphasia or cognitive impairment, as determined by the clinical team. Sample size was calculated with the Power Analysis and Sample Software (PASS) for our primary objective. Power analysis for a two-tailed linear regression model with α ≤ 0.05, β 0.80, and an effect size of 0.15 resulted in a required sample size of 73. An expected attrition rate of 20% resulted in a sample size of 88, with the intent to enroll 44 males and 44 females. This study has been approved by the internal review boards of both affiliated universities, and written informed consent will be obtained from all participants prior to study enrollment.

### Data collection

Quantitative data will be collected *via* electronic medical record (EMR) review and participant self-report. Study data will be collected and managed using REDCap electronic data capture tools (39). Clinical data collected *via* EMR will include date and type of stroke, inferred Modified Rankin Scale, and screening for anxiety (Generalized Anxiety Disorder-7) and depression (Patient Health Questionnaire-9). Baseline data collection will occur at the first post-stroke clinic visit (within 8 weeks of stroke event) *via* paper, verbal, or electronic surveys. Study personnel will contact participants *via* telephone, email, or text message at 3 months post-stroke to identify participant interest in focus group participation and to remind participants of upcoming 6 month data collection. Follow-up data collection will occur at 6 months post-stroke, either during the 6 month clinic visit or *via* email or telephone.

Qualitative data will be collected *via* small focus group interviews led by 3 nurse investigators (ML, AB, and SC). The interviews will occur either face-to-face or *via* secure electronic platform (i.e., Zoom or Microsoft TEAMS), depending on the local and national guidelines related to the COVID-19 pandemic at the time of the interviews. Semi-structured interview guides will be used, and field notes will be gathered during the interviews. Interviews will be audio recorded (face-to-face) or audio and video recorded (electronic platform). All study participants have the option to participate in a focus group within 9 months of their stroke. Interested participants will be contacted to confirm availability for scheduled interviews. This initial convenience sampling will evolve to purposive sampling to support inclusion of participants from both sexes and various age groups. We anticipate data saturation will be reached after 2 to 3 focus group interviews.

### Measures

#### Baseline

The exposure variables for this study are experiences of racism and resilience. Experiences of racism are measured with the Everyday Discrimination Scale (EDS) and the Heightened Vigilance Scale. The EDS, a 10-item instrument developed to measure day-to-day experiences of discrimination, has demonstrated evidence of construct validity (confirmatory factor analysis = 1 factor) in Black populations (40). The Heightened Vigilance Scale is a 4-item instrument developed from ethnographic research about how participants anticipated and prepared for racial discrimination (41, 42). Resilience is measured with the Brief Resilience Scale (BRS) and the Connor-Davidson Resilience Scale 10 (CD-RISC 10). The BRS, a 6-item instrument developed to measure resilience as the ability to “bounce back” from stress, has demonstrated excellent evidence of convergent, divergent, and construct validity (principal component analysis = 1-factor solution) (43). The CD-RISC 10 is a 10-item version of the 25-item CD-RISC, which was developed to measure resilience as a stress coping ability (44). The CD-RISC 10 has demonstrated convergent and divergent validity, and good to excellent agreement with the original CD-RISC scale (45).

Covariates include sociodemographic and psychosocial variables. Self-report sociodemographic variables will include age, sex, marital status, education, income, health insurance status and type, employment status, number of people living in the household, and neighborhood (residential address). Pandemic-related stress is measured with an adapted version (13 items) of the 23-item COVID-19 Stressors Scale, which has excellent evidence of convergent, divergent, and construct validity (exploratory factor analysis = 1 factor) (46). Psychological stress is measured with the 10-item Perceived Stress Scale (PSS), which measures perceived stress in the past month (47). Mindfulness is measured with the Cognitive and Affective Mindfulness Scale-Revised (CAMS-R), a 12-item instrument developed with a focus on mindfulness as the ability to regulate attention and orient oneself, with awareness and acceptance, toward the present experience (48). The CAMS-R has demonstrated adequate evidence of convergent and divergent validity (48). All survey instruments demonstrate adequate evidence of internal consistency reliability (Cronbach's alpha 0.72–0.96) (42–44, 46–49).

#### Follow-up

The World Health Organization Quality of Life BREF (WHOQOL-BREF) scale is used to operationalize quality of life, the outcome variable for this study. The WHOQOL-BREF, a 26-item version of the WHOQOL-100 instrument, has domains for physical, psychological, social and environmental quality of life (QoL). This measure has demonstrated adequate evidence of internal consistency (Cronbach's α > 0.7), discriminant validity, and construct validity in a large, cross-cultural sample (>11,000) of adults from 23 countries (50). The WHOQOL-BREF has also demonstrated adequate evidence of reliability and validity in a sample of 4,628 adults with various diseases or health conditions, including stroke, cardiovascular diseases, and neurodegenerative disease (51).

### Data analysis

Descriptive statistics will be computed for the sociodemographic variables. Means and standard deviations will be used to describe continuous variables, and frequencies and percentages will be used to describe categorical variables. Internal consistency reliability for the exposure, outcome, and psychosocial variables will be computed with Cronbach's alpha. Confirmatory factor analysis will be used to evaluate the construct validity of the BRS and CD-RISC 10 instruments. The effect of the primary exposure variable (experiences of racism) on the outcome variable (QoL) will be evaluated with multiple-level linear models. The intersection of sex and race will be evaluated by including sex as a covariate in the initial model. Sociodemographic, clinical and psychosocial variables with *p* ≤ 0.10 significance for bivariate association with QoL will be considered for model inclusion. Resilience will be added to the final model to test the moderating effect of resilience on the relationship between racism and QoL. The direct effect of resilience on QoL will be assessed with multiple-level linear models, with the same parameters for the modeling of the effect of racism on QoL. The study statistician will use SPSS software (version 28) to perform the quantitative data analysis (52).

Qualitative data will be de-identified, transcribed verbatim and exported into NVivo software (QSL International) for coding and analysis. Inductive and deductive methods will be used to develop codes and emerging themes. We will use qualitative descriptive analysis methods for content thematic analysis. Thematic analysis will continue until the 3 nurse researchers (ML, AN, SC) reach consensus for the final themes. The Consolidated Criteria for Reporting Qualitative Research (COREQ) checklist will be utilized throughout the qualitative process to ensure validity and rigor (53).

## Discussion

Stroke is a sudden, life-threatening stressor that challenges survivors. For some, stroke can be a wake-up call that results in a reordering of priorities and refocusing on what is truly important to the survivor. Other SS can feel overwhelmed, resulting in considerable psychological distress and negative impacts on recovery. Failure to recognize and address the emotional and psychological responses to acute stroke is an often unmet need, especially for survivors of milder stroke. This can negatively impact stroke-survivor outcomes, including quality of life. In addition to the stress from acute stroke, Black SS experience racism, a major stressor with both acute and chronic effects on health. Thoughtful consideration of the study context and target population informed the selection of study variables and methods used to measure these variables. This study will be initiated in the context of nationwide protests and calls for social justice in the aftermath of police killings of Black Americans (e.g., George Floyd, Breonna Taylor, and others). Additionally, participant recruitment will begin during the COVID-19 pandemic. These contexts influenced measures for experiences of racism, the primary exposure variable of the study, and for psychosocial covariates related to stress. Experiences of racism, which include prejudice and discrimination, are measured with scales for both discrimination and vigilance. Vigilance is a type of anticipatory stress that has been associated with increased risk for hypertension in Black people (41). During the pandemic, vigilance has been associated with depressive symptoms (β = 0.90, 95% CI 0.12–1.67) and anxiety (β = 1.64, 95% CI 0.82–2.45) in Black Americans (54). The study context also informed inclusion of covariates for perceived stress, recognized as being independently associated with stroke (3), and more likely to be identified as a stroke risk factor by Black Americans (55) and stress related to the COVID-19 pandemic, which has disproportionally affected Black Americans compared to White Americans (56).

Sociodemographic variables have been operationalized to facilitate an accurate assessment of specific social determinants of health that intersect with structural racism. One example of structural racism is residential segregation, which influences socioeconomic status through reduced access to quality education, employment opportunities, and health care (18, 19). Residential address will be used to determine the neighborhood segregation index for the participant's household. Annual income categories (USD) begin at “less than $10,000” and increase incrementally over 8 categories to “over $75,000.” This method of measuring income, combined with the number of persons living in the household, allows for assessment of participant household income in relation to the current USA poverty threshold. Black Americans are more likely to live in poverty, tend to have lower education levels, and reduced access to preventive health resources than other racial groups (57). Black Americans in middle or upper socioeconomic classes have poorer health outcomes than non-Hispanic White Americans in the same classes, potentially due to multiple stressors including racism (57).

Quality of Life is operationalized with the WHOQOL- BREF because the scale was deemed a better fit for our study purpose and target population than scales focused specifically on stroke. The aim of our study is to examine the effects of racism (a major stressor) and resilience on QoL for Black SS in the first 6 months of recovery from stroke. The four domains of the 26-item WHO-QoL BREF scale (physical, psychological, social, environmental) reflect concerns specifically relevant for our target population. The 7 items in the physical domain capture the most germane physical health concerns (pain, energy, sleep, mobility, activities, medication, and work) for this study population. Included in the psychological domain are both positive and negative feelings and self-esteem, as well as items more specifically applicable to SS such as cognition and body image. The Black church is well known as a source of social support for Black Americans (58), who tend to be more religious than Americans of other racial groups (59). The psychological domain includes an item on spirituality, and the 3-items in the social domain focus on interpersonal relations and social support. Lastly, the 8-items in the environmental domain reflect satisfaction with finances, living conditions, access to health services and transport, and feelings of safety and security, all factors related to SDOH shaped by structural racism in the USA (18).

While the Stroke Impact Scale (SIS) demonstrates adequate reliability and validity in the stroke population (60), 4 of the 8 subscales in the instrument focus on physical aspects of stroke recovery (physical problems, activities of daily living, mobility, hand function), which are not a focus of this study. A study of SS in the chronic phase of recovery found statistically significant associations between perceived stress (PSS) and the SIS subscales for mood and emotion, participation/role function, and memory and thinking, and between resilience (BRS) and SIS subscales for mood and emotion, participation/role function and communication (61). Of interest, there were no significant associations between either stress or resilience with physical outcomes of the SIS (61). Additionally, a recent study of SS in the acute phase of recovery found no statistically significant associations between resilience (measured during hospitalization for stroke) and activities of daily living, measured 3 months after stroke with the Barthel Index (62). The authors concluded that these results may relate to the mild-to-moderate stroke severity and hence higher-level functioning of their sample, or that resilience may be associated with psychosocial “adversity” rather than physical “adversity” (62).

Determinants of resilience vary between individuals and communities; fostering resilience by promoting healthy communities also supports individual resilience (26). Consideration of the cultural context, including culturally-related goals and access to resources, is central to consideration of individual-level resilience (26). The cultural history of Black people in the USA has, by necessity, centered on overcoming obstacles against all odds, linking individual health to that of the community, and focusing on the power of spirituality (63). Thus, the ability of Black Americans to survive, and even thrive, in the context of racism suggests both individual and collective resilience (64).

Few studies have examined resilience or resilience factors in Black people either at risk for or diagnosed with cardiovascular disease. Among Black participants in the Women's Health Initiative study, higher resilience was associated with lower stress, higher education, less obesity, less use of lipid-lowering medications, and higher levels of physical activity (65). The Morehouse-Emory Cardiovascular Center for Health Equity study (MECA) examined neighborhood perceptions and psychosocial characteristics as resources for resilience to cardiovascular disease among Black residents in neighborhoods with lower vs. higher risk of adverse cardiovascular outcomes (66). Residents in neighborhoods with lower risk of cardiovascular disease (CVD) reported better neighborhood characteristics (increased odds of higher neighborhood aesthetics and access to healthy foods, and decreased odds of neighborhood violence) and better psychosocial characteristics (increased odds of higher optimism and higher purpose in life, and lower odds of depressive symptoms). Resilient coping was not a significant factor in the analysis, and the odds for optimism and depressive symptoms were attenuated by individual socioeconomic status (66). Religious beliefs and behaviors, mindfulness, and emotional support may buffer the effects of racism on health outcomes in Black people, but more research is needed (67).

Existing evidence on differences in coping with stress between Black men and women led us to include the interaction of sex with race in our statistical modeling for this study (68–70). In consideration of the relative lack of studies focused on resilience in Black patients with stroke or other CVD, psychometric validation of the two resilience scales in this study (BRS, CD-RISC 10) will provide guidance for measuring resilience in future studies. Mindfulness, which is positively associated with resilience (29, 30), focuses on orienting oneself to the present experience with awareness and acceptance of the experience (48). Mindfulness facilitates emotion regulation, reducing responses such as avoidance, denial, worry, and elevated symptoms of distress related to either under- or over-engagement with emotions (48).

The mixed-methods design of the study facilitates a holistic view of the effects of experiences of racism and resilience on Black SS QoL. In addition to the quantitative measures of the participants, focus group interviews will capture a meaningful qualitative aspect. The participants' spoken words about their experiences of racism and resilience will provide valuable insight into how these experiences impact their quality of life post-stroke. Open-ended interview questions have been carefully developed by the researchers to actively engage participants to discuss specifically their experiences of racism, what resiliency means to each participant, and how racism and resiliency affected their quality of life post-stroke. We expect this qualitative data will enrich quantitative survey data and inform the development of interventions for Black SS to manage stressors, including racism, that impact quality of life after stroke. As a dynamic process affected by a variety of factors, resilience can be considered modifiable and amenable to change through intervention. Identification of resilience resources for specific populations (e.g., Black SS) could inform interventions to improve outcomes (e.g., QoL) by enhancing resilience. Potential targets for resilience interventions include social support (71), the narrative process of telling one's story regarding stroke and stroke recovery (25, 72), and stress reduction through mindfulness (57).

## Ethics statement

The studies involving human participants were reviewed and approved by University of Texas Health Science Center Houston Committee for the Protection of Human Subjects and University of Houston Internal Review Board. The patients/participants provided their written informed consent to participate in this study.

## Author contributions

ML and AS contributed to the conception and design of the study. AC organized and managed the database in REDCap. AC, GC, and MO contributed to the acquisition of data for the work. ML, SC, and AB contributed to the implementation plan for the qualitative portion for the study. ML wrote the first draft of the manuscript. All authors contributed to manuscript revision, read, and approved the submitted version.

## Funding

This project was funded by University of Houston Grants to Enhance Research on Racism, PROJECT 5999-H0586-B2080-C0505812 (67294), Award 000180960. This funding opportunity was initiated by Dr. Renu Khator, President of University of Houston, to enhance research on racism. Funds for open access publication fees were provided through new faculty start-up funds provided to Dr. Mary Love upon her employment with University of Houston in 2019.

## Conflict of interest

The authors declare that the research was conducted in the absence of any commercial or financial relationships that could be construed as a potential conflict of interest.

## Publisher's note

All claims expressed in this article are solely those of the authors and do not necessarily represent those of their affiliated organizations, or those of the publisher, the editors and the reviewers. Any product that may be evaluated in this article, or claim that may be made by its manufacturer, is not guaranteed or endorsed by the publisher.
